# Ting JY: Letter to the Editors: The potential role of prehospital thrombolysis and time-critical stroke transfers in the northern Norway aeromedical retrieval system

**DOI:** 10.1186/1865-1380-4-43

**Published:** 2011-07-18

**Authors:** Jan Norum

**Affiliations:** 1 Northern Norway Regional Health Authority trust, N-8038 Bodø, Norway; 2 Department of Clinical Medicine, Faculty of Health Sciences, University of Tromsø, N-9037 Tromsø, Norway; 3 Department of Oncology, University Hospital of North Norway, N-9038 Tromsø, Norway

## 

In our study [1], we reported a lengthy 3 h 33 min one-way transfer time from the Norwegian Arctic to the mainland. However, I fully agree with Ting that we should have focused more on the present system where ECGs may be communicated digitally from the ambulances, district medical centers and local hospitals to cardiologists at the main hospitals. This was however mentioned in our previous article on ambulances [2]. In practice, patients in the Svalbard Islands are now transported to the small hospital unit in Longyearbyen for diagnosis (ECG) and initial treatment. The ECG is then communicated digitally to the cardiologist on duty at the University Hospital of North Norway (UNN) located on the mainland (city of Tromsø). The recognition of STEMI can be achieved and advice concerning prehospital thrombolysis given by the cardiologist prior to the lengthy transfer to the mainland. In some settings ECGs may also be communicated from minor bases in the Arctic, but be aware of communication problems this far to the north [3]. I am aware of one case where an ECG was sent by fax from a remotely located base (Jan Mayen) in the Norwegian Arctic to the UNN.

Concerning neurological/stroke patients, there is at present intense debate about whether the small hospital unit at Longyearbyen on the Svalbard Islands should be equipped with a CT scanner. Such a tool would make diagnosis of patients suitable for thrombolysis possible. However, such an investment will not be cost-effective with a low volume of patients and must be considered only from a preparedness point of view. I agree with Ting that it is uncertain whether more Arctic tourism will increase the aeromedical workload. However, the increasing activity with regard to oil and gas installations together with shipping should also be considered in this setting [3].

## Competing interests

The author declares that they have no competing interests.
